# Schwannoma of the thyroid bed: A case report and review of the literature

**DOI:** 10.1097/MD.0000000000018814

**Published:** 2020-01-31

**Authors:** Ji Yun Kang, Kyung Sik Yi, Sang-Hoon Cha, Chi-Hoon Choi, Yook Kim, Jisun Lee, Seung-Myoung Son

**Affiliations:** a Department of Radiology; b College of Medicine and Medical Research Institute; c Department of Pathology, Chungbuk National University Hospital, Cheongju, Republic of Korea.

**Keywords:** Core needle biopsy, Fine needle aspiration, Schwannoma, Thyroid

## Abstract

**Rationale:**

Schwannomas involving the thyroid gland are very rare and only a few cases have been reported in the literature. However, previous reports did not distinguish between thyroid bed schwannomas and intrathyroidal schwannomas. Here, we report a thyroid bed schwannoma mimicking a malignant thyroid nodule and review the literature on thyroid bed schwannomas.

**Patient concerns:**

A 33-year-old woman presented at our hospital with mild neck swelling.

**Diagnosis:**

Thyroid ultrasound revealed a well-defined, oval-shaped, markedly hypoechoic solid nodule with echogenic foci suggesting macro- and microcalcifications in the left thyroid gland. The lesion was considered a “highly suspicious” intrathyroidal nodule, based on the guidelines for the assessment of thyroid nodules. Fine needle aspiration was performed twice, but the cytological results were nondiagnostic.

**Interventions:**

Left thyroidectomy was performed, and schwannoma of the thyroid bed was confirmed on histopathologic analysis.

**Outcomes:**

The patient was in a stable condition after surgery, and the thyroid function test results were within the normal range.

**Lessons:**

Diagnosis of a schwannoma of the thyroid bed is challenging because its incidence is extremely low, and it is often misdiagnosed as an intrathyroidal nodule on ultrasonography. Therefore, it is advisable to adopt a diagnostic strategy to perform additional core needle biopsy in cases of thyroid nodules with nondiagnostic fine needle aspiration results and to consider the location of the lesion more carefully to determine the suitable therapy.

## 1 Introduction

Schwannomas are slow-growing benign tumors that may arise from Schwann cells in the nerve sheath anywhere in the body. About 25% to 45% of schwannomas occur in the head and neck region, but schwannomas of the thyroid bed are extremely rare. Additionally, they are difficult to differentiate from thyroid nodules because their sonographic features and clinical symptoms are similar.^[1]^ The thyroid gland itself is a rare location for schwannomas; few cases have been reported in the literature, and those reports included cases of intrathyroidal schwannomas and thyroid bed schwannomas.^[2–17]^ Here, we report the case of a thyroid bed schwannoma that was confirmed by left thyroid lobectomy. Its marked hypoechogenicity and the presence of echogenic foci on ultrasonography suggested macro- and microcalcifications, which raised suspicion of a malignant thyroid nodule.

## 2 Case report

### 2.1 Patient information and clinical findings

**Figure 1:**
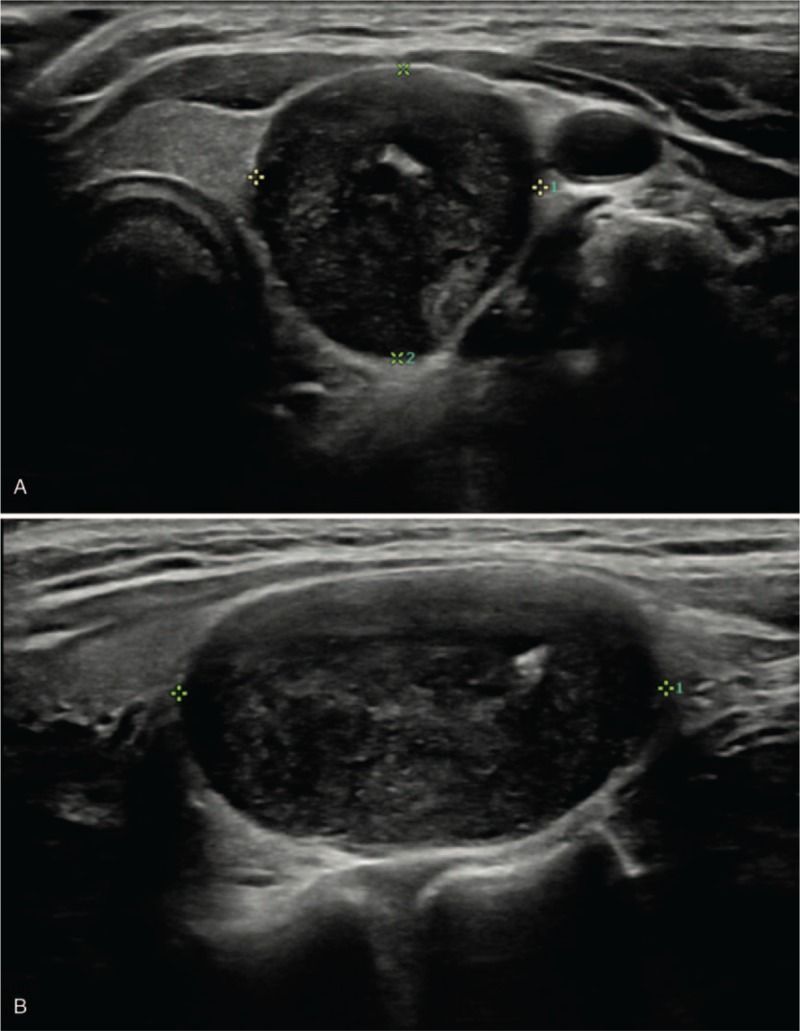
A 33-year-old woman presented with a schwannoma of the thyroid bed. Transverse (A) and longitudinal (B) ultrasound images showed an approximately 1.7 cm sized, well defined, markedly hypoechoic solid nodule with echogenic foci (arrowhead) suggesting macro- and microcalcifications and smooth borders (arrow). It appeared to be an intrathyroidal nodule with high suspicion of malignancy.

A 33-year-old woman presented to an endocrinology clinic with a palpable neck mass that had lasted for 2 years. Results of a thyroid function test were normal, and no other symptoms were present. Ultrasonography of the mass was performed, revealing a well-defined, oval-shaped, markedly hypoechoic intrathyroidal nodule 3 cm in size with echogenic foci suggesting macro- and microcalcifications in the left thyroid lobe (Fig. 1). Because of the sonographic findings, it was considered highly suspicious for malignancy.^[18]^

Ultrasound-guided fine needle aspiration (FNA) was performed, and the cytological results were nondiagnostic. Therefore, according to the guidelines,^[18]^ repeat FNA was performed, but the results were nondiagnostic again.

### 2.2 Therapeutic interventions

**Figure 2:**
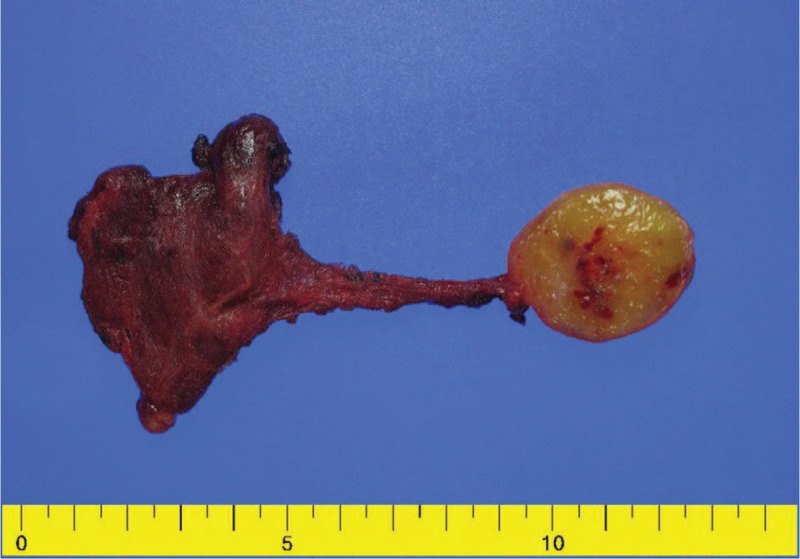
The surgical specimen after lobectomy of the left thyroid gland exhibited an extrathyroidal yellowish mass, measuring 1.7 × 1.7 × 1.3 cm, abutting to the left thyroid gland. No abnormality was found in the left thyroid gland.

Because of the suspicion of malignancy on ultrasonography, left thyroid lobectomy was performed. A section removed intraoperatively was frozen, and an analysis was performed, revealing a benign spindle cell tumor. Macroscopic findings revealed a yellowish mass adherent to the left thyroid lobe that was about 1.7 × 1.7 × 1.3 cm in size. The left thyroid lobe showed no abnormality (Fig. 2). The patient was in a stable condition after surgery, and the thyroid function test results were within the normal range.

### 2.3 Pathologic findings

**Figure 3:**
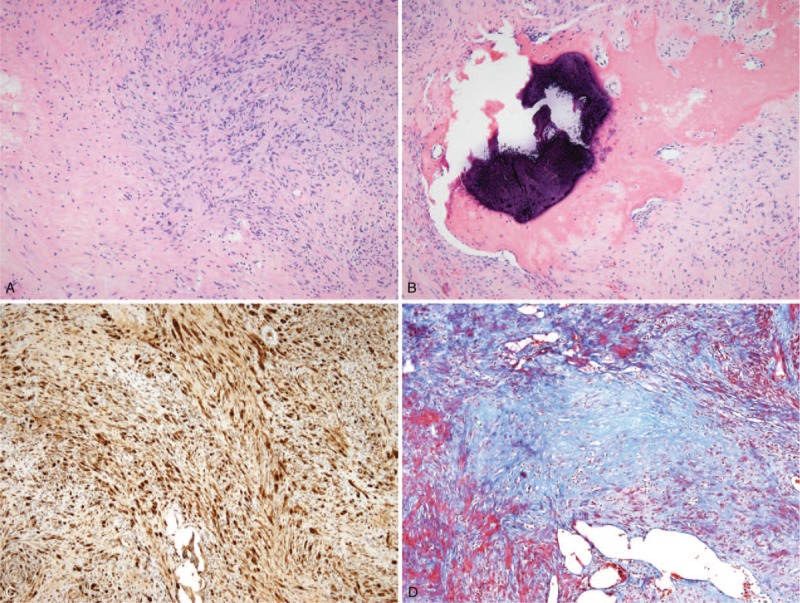
Histopathological examination of the surgical specimen. (A) Histopathological examination of the surgical specimen revealed a schwannoma, composed of compact areas of spindle cells (Antoni A, arrowheads) and loosely arranged foci (Antoni B, asterisk). (B) A small calcification and ossification (arrow) were observed. (C) Immunohistochemistry shows positive staining for the S-100 protein, confirming the diagnosis of schwannoma. (D) In this Masson trichrome-stained section, collagen deposition was revealed as blue areas.

A histopathological examination of the surgical specimen revealed a schwannoma in the perithyroid tissue, composed of compact areas of spindle cells (Antoni A) and loosely arranged foci (Antoni B) (Fig. 3A). A small area of calcification was observed (Fig. 3B), corresponding to the macrocalcification observed on ultrasonography. Positive immunohistochemical staining for the S100 protein confirmed the diagnosis of schwannoma (Fig. 3C). A Masson trichrome-stained section revealed an area of collagen deposition (Fig. 3D).

### 2.4 Ethical considerations

The institutional review board approved a retrospective review of the medical records and waived the requirement to obtain informed consent.

## 3 Discussion

Schwannomas are slow growing, encapsulated tumors originating from neuronal sheath cells, such as Schwann cells. Although schwannomas of the head and neck region are common, schwannomas involving the thyroid gland are particularly rare.^[1]^ Some cases have been reported in the literature, including cases of intrathyroidal schwannomas and thyroid bed schwannomas.^[2–17]^ Primary thyroid schwannomas are thought to originate from the intrathyroid sensory nerves or from autonomic innervation to the thyroid.^[17]^ In contrast, schwannomas in the thyroid bed may arise from the sympathetic chain, glossopharyngeal, vagus, and accessory, recurrent laryngeal, or hypoglossal nerves.^[4,14,16,17]^ Unlike intrathyroidal schwannomas, thyroid bed schwannomas can be resected without the need for a thyroidectomy.^[17]^ However, thyroid bed schwannomas can appear as intraglandular lesions on ultrasonography, ^[12,14,16,17]^ as in this case, and it has been reported that magnetic resonance imaging can be helpful for identifying the tumor's origin.^[16]^ Nevertheless, previous reports did not distinguish thyroid bed schwannomas and intrathyroidal schwannomas. To aid our understanding of the case, we have performed a search of the literature using the terms “schwannoma” and “thyroid gland”, and we reviewed all cases previously published in English related to thyroid bed schwannomas only (Table 1).

**Table 1:**
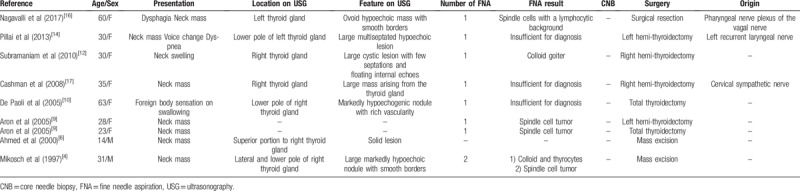
Summary of thyroid bed schwannoma cases published in the literature.

Sonographic findings describe schwannomas as well-defined, round or ovoid, with a thickened wall, and abundant internal and peripheral vascularization. Schwannomas can show echogenicity of varying degree on ultrasonography, according to the composition of Antoni cells and cystic changes, but they generally exhibit hypoechogenicity similar to or lower than that exhibited by muscle tissue.^[10,19]^ Calcification in a schwannoma is extremely rare, which can make it difficult to establish a differential diagnosis. Calcification is a normal finding related to degenerative changes, and only a few reports have described cases of calcification in schwannomas.^[20–22]^ Histopathological examination of our case revealed a small calcification, corresponding to the macrocalcification observed on ultrasonography. However, microcalcifications were not observed upon microscopic assessment. Echogenic foci observed on ultrasonography, except for macrocalcification, might be due to collagen deposits in schwannomas, which, in turn, might correlate with findings from histologic examination of Masson trichrome-stained sections.^[19]^ In our case, differential diagnosis was complicated by the tumor's marked hypoechogenicity and the presence of echogenic foci that suggested macro- and microcalcifications, which led to a suspicion of malignancy.^[18]^ To the best of our knowledge, a thyroid bed schwannoma mimicking a malignant thyroid nodule has not been reported in the literature to-date.

FNA is easily available and accurate for most head and neck masses; however, for the diagnosis of schwannoma, the sensitivity of FNA is relatively low, at 0% to 40%, with unsatisfactory specimen rates for FNA of 36% to 50%.^[23,24]^ This limitation of FNA is due to the histologic characteristics of schwannomas, such as their dense interstitial components, hypocellular Antoni B areas, and frequent cystic degeneration.^[23]^ For thyroid bed schwannomas, the results of FNA for 4 cases reported in the literature were nondiagnostic, and for 4 other cases suggested only spindle cell lesions.^[4,6,9,10,12,14,16,17]^ Recently, core-needle biopsy (CNB) has been proposed as a complementary method for FNA, and some studies that compared it with FNA concluded that CNB provided a higher diagnostic accuracy with less invasive histological and immunohistochemical characterization of many head and neck tumours, especially schwannomas.^[25,26]^ In our case, repeated FNA was performed due to the nondiagnostic result obtained with the initial FNA, but no further CNB was performed. CNB could have been used as a method to determine the appropriate preoperative treatment strategy and make an accurate diagnosis for this patient, whose FNA results were inconclusive.

In conclusion, we report a case of a thyroid bed schwannoma, the diagnosis of which was made by left thyroid lobectomy due to the tumor's marked hypoechogenicity and the presence of echogenic foci that were suggestive of macro- and microcalcifications on sonographic findings, which, in turn, lead to a high suspicion of a malignant thyroid nodule. Diagnosis of a schwannoma of the thyroid bed is challenging because its incidence is extremely low and it is often seen as an intrathyroidal nodule on ultrasonography. Therefore, it is advisable to have a diagnostic strategy to perform additional CNB in cases of thyroid nodules with nondiagnostic FNA results and to consider the location of the lesion more carefully in order to determine the proper therapy.

## Author contributions

**Conceptualization:** Kyung Sik Yi, Jisun Lee, Yook Kim, Ji Yun Kang.

**Resources:** Kyung Sik Yi, Seung-Myoung Son.

**Data curation:** Kyung Sik Yi, Ji Yun Kang.

**Formal analysis:** Kyung Sik Yi, Ji Yun Kang.

**Supervision:** Sang-Hoon Cha, Kyung Sik Yi, Chi-Hoon Choi.

**Writing – original draft:** Kyung Sik Yi, Ji Yun Kang.

**Writing – review & editing:** Kyung Sik Yi, Ji Yun Kang.

Kyung Sik Yi orcid: 0000-0002-4274-8610.
